# Larger Resin Ducts Are Linked to the Survival of Lodgepole Pine Trees During Mountain Pine Beetle Outbreak

**DOI:** 10.3389/fpls.2019.01459

**Published:** 2019-11-26

**Authors:** Shiyang Zhao, Nadir Erbilgin

**Affiliations:** Department of Renewable Resources, University of Alberta, Edmonton, AB, Canada

**Keywords:** climate change, Dendroctonus ponderosae, dendroecology, insect outbreaks, natural disturbances, *Pinus contorta* var. latifolia, resin duct, tree survival probability

## Abstract

Periodic mountain pine beetle outbreaks have killed millions of hectares of lodgepole pine forests in western North America. Within these forests some pine trees often remain alive. It has been rarely documented whether anatomical defenses differ between beetle-killed and remaining live pine trees, especially at the northern latitudinal range of beetles in North America. In this study, we compared the resin duct-based anatomical defenses and radial growth between beetle-killed and live residual lodgepole pine trees, and we characterized the resin ducts and the growth of the residual trees before and after outbreak. We found that tree radial growth was not associated with tree survival. The best two predictors of tree survival were resin duct size and production (number per year). Trees having larger but fewer resin ducts showed higher survival probability compared to those with smaller but more abundant resin ducts annually. Residual trees had larger resin ducts prior to the outbreak and continued having so after the outbreak. We further categorized residual trees as healthy (having no signs or symptoms of insect or pathogen attacks), declining (with signs or symptoms of biotic attacks), and survived (from mountain pine beetle attacks during the outbreak) to investigate resin duct-based anatomical defenses among them. Healthy trees had consistently larger resin ducts than declining trees in the past 20 years in post-outbreak stands. Survival trees ranked between healthy and declining trees. Overall, these results demonstrate that resin duct size of lodgepole pine trees can be an important component of tree defenses against mountain pine beetle attacks and suggest that lodgepole pine trees with large resin ducts are likely to show resistance to future bark beetle attacks.

## Introduction

Forest ecosystems have coevolved with natural disturbances such as wildfire, windthrow, and pest and pathogen outbreaks. These normative disturbances consistently shape forest ecosystem and functions (Mattson, 1996). However, climate change has altered natural disturbance regimes and in most cases increased frequency and severity of insect outbreaks across many forest types (Logan et al., 2003; Kirilenko and Sedjo, 2007; Bentz et al., 2010; Raffa et al., 2017; Montoro Girona et al., 2018). In North America, periodic mountain pine beetle (MPB, *Dendroctonus ponderosae* Hopkins, Coleoptera: Curculionidae) outbreaks had historically occurred in the western United States and Canada (British Columbia). Recently, however, outbreaks have shifted to higher elevations (Cudmore et al., 2010; Raffa et al., 2017) and naïve habitats in northern latitudes and eastern longitudes (Cullingham et al., 2011; Erbilgin et al., 2014), which were originally considered to be climatically not suitable to the beetles. The most recent MPB outbreaks in Canada have affected over 18 million hectares of pine forests, mainly lodgepole pine trees (*Pinus contorta* Douglas ex Loudon var. *latifolia* Engelm. ex S. Watson) (Natural Resources of Canada, 2019). Such outbreaks caused substantial changes in above- and below-ground biotic communities and abiotic conditions (Griffin and Turner, 2012; Hawkins et al., 2013; McIntosh and Macdonald, 2013; Treu et al., 2014; Karst et al., 2015; Pec et al., 2017). Some individual trees survived these outbreaks in some stands, resulting in a wide range of tree mortality from 0% to 100% (Erbilgin et al., 2017; Six et al., 2018). Although studies have suggested that both chemical and anatomical defenses have possible roles in the survival of trees under a high density of bark beetle attacks (Kane and Kolb, 2010; Ferrenberg et al., 2014; Hood et al., 2015; Erbilgin et al., 2017), the anatomical characteristics determining the survival of these pine trees are less understood.

Conifer trees have developed constitutive and inducible defense mechanisms that integrate both anatomical structures and toxic chemicals against bark beetle attacks (Franceschi et al., 2005; Erbilgin et al., 2006; Keeling and Bohlmann, 2006; Kolosova and Bohlmann, 2012; Erbilgin, 2019). During the initial host colonization by bark beetles, constitutive defenses are the first line of defense that can substantially lower the probability of successful beetle colonization. However, if constitutive defenses are not successful in ceasing the continuous beetle attacks, additional induced defenses are activated to protect the trees from bark beetle colonization. Toxic and sticky resins are the primary constituents of both the constitutive and induced defenses of conifers. As a part of anatomical defenses, resin ducts are responsible for the production, storage, and translocation of resins to the site of beetle entry and can be found in both the secondary phloem and xylem in pines (Franceschi et al., 2005). Similar to the chemical defenses, resin ducts can also be induced in pines by beetle attacks (Hudgins et al., 2004).

Historically, studies on pine–bark beetle interactions have mainly focused on tree chemical defenses (e.g., Erbilgin et al., 2017; Raffa et al., 2017; Celedon and Bohlmann, 2019; Erbilgin, 2019), while the role of anatomical defenses, particularly resin duct characteristics, in pine defenses against bark beetles has received relatively less attention (Kane and Kolb, 2010; Ferrenberg et al., 2014; Gaylord et al., 2015; Hood and Sala, 2015; Mason et al., 2019). In contrast to the chemical defenses, resin ducts are embedded in the annual rings of tree xylem, providing valuable information regarding the history of tree defenses (Mason et al., 2019). Earlier studies reported differences in the anatomical defenses of beetle-killed and residual pine trees (Kane and Kolb, 2010; Ferrenberg et al., 2014). Hood and Sala (2015) found that increasing resin flow was positively related to resin duct characteristics and that resin ducts could be indicators of enhanced tree resistance against bark beetles. However, similar studies are currently lacking in the newly expanded range of MPB in Alberta.

Mountain pine beetle outbreaks have altered forest stand conditions, including soil moisture, soil nutrients, soil microbial communities, and aboveground plant communities (Griffin and Turner, 2012; Hawkins et al., 2013; McIntosh and Macdonald, 2013; Pec et al., 2015; Pec et al., 2017), and, in many cases, these changes have lasted for years (Cigan et al., 2015; Karst et al., 2015; Dhar et al., 2016). Earlier studies examined the radial growth rate of residual overstory pine trees as a perimeter of tree health after outbreaks and reported accelerated radial growth in the historical range of MPB (Murphy et al., 1999; Alfaro et al., 2010; Amoroso et al., 2013; Hawkins et al., 2013). However, it is hard to predict how naïve lodgepole pine trees in Alberta have responded to the altered stand conditions caused by beetle outbreaks. Specifically, the consequences of MPB outbreaks on the anatomical defenses and radial growth of residual lodgepole pine trees remain to be investigated.

In this study, we focused on the recent MPB outbreak in the beetle’s most northern range in the lodgepole pine forests of western Canada. We compared the growth rate and resin duct characteristics between beetle-killed and residual pine trees to determine the role of growth and defenses in tree survival under high intensity bark beetle attacks. We hypothesize that residual pine trees would have more or larger anatomical defense structures but lower radial growth compared to MPB-killed trees as residual trees might allocate more resources towards defense rather than growth. Furthermore, we investigated whether post-outbreak stand conditions altered the historical radial growth and resin duct defenses by the comparing growth rate and resin duct characteristics of residual pine trees between pre- and post-outbreak periods. Considering the reduced competition among overstory pine trees in the post-outbreak stands, we hypothesize that residual pine trees would produce more carbon resources, which may lead to a greater allocation to anatomical defense but can cause delayed growth release after MPB outbreak, compared to before outbreak. Finally, we tested whether radial growth and resin duct defenses varied among different categories of residual pine trees for the most recent 20 years of growth. We hypothesize that healthy residual pine trees would grow faster and have stronger anatomical defenses than trees showing symptoms of declining.

## Materials and Methods

### Study Area Description

Initially, we used provincial MPB mortality maps to select sites showing various levels of pine mortality in western Alberta (Government of Alberta, 2015). After the initial selection, on the ground, we only selected sites that mature lodgepole pine trees (dead or alive) over 15 cm at diameter breast height (DBH) constituted more than 50% of overstory canopy trees (**Table S1**). This process yielded a total of 31 sites (**Figure S1**). In these sites, lodgepole pine mortality due to MPB ranged from 2% to 83% (**Table S2**). All sites were located in lower and upper foothills, dry mixedwood, and lower boreal highlands natural subregions. The elevation of these sites ranged from 600 m to 1,000 m and the latitude ranged from 54.43256 to 56.84269. In each site, we set up two plots (each 20 m x 20 m) showing different percentages of pine mortality, and the plot centers were at least 100 m away from each other. We confirmed trees killed by MPB by the signs of beetle attacks, including pitch tubes, brood beetle emergence holes, and extensive beetle galleries under the bark (Erbilgin et al., 2017). We considered all live mature lodgepole pine trees in each plot as residual trees and, based on their apparent health conditions, classified them into three categories as healthy, declining, and survived. Healthy trees showed no obvious symptoms of any pathogen or insect attacks. Declining trees had symptoms and signs of insect, other than MPB, and pathogen attacks with dying branches, bark lesions, sparse crown, and yellow-red needles. Survived trees were healthy but with symptoms of unsuccessful MPB attacks. We determined unsuccessful MPB attacks on survived trees based on the failed beetle reproduction, i.e., presence of short maternal galleries, absence of oviposition and larval galleries, and brood emergence holes (Erbilgin et al., 2017).

### Sample Collection and Preparation

In each plot, we randomly selected up to three MPB-killed and six residual (two from each of the three categories) trees from the dominant or codominant crown class. Since some plots did not have trees in all three residual tree categories, the number of trees selected in each category varied among plots. In total, we selected 140 beetle-killed and 210 residual (76 healthy, 62 declining, and 72 survived) trees. We cored the selected residual trees once at DBH on the south aspect using a 12 mm increment borer in May 2016, and, thus, the last complete ring formed on the cores occurred in 2015. From beetle-killed trees, we took a single wedge at the same height and aspect as the residual trees. Each wedge contained at least 15 years of growth counting back from the year of death. In addition, we recorded the DBH of all residual pine trees sampled. However, we could not measure the DBH of all beetle-killed trees because their bark had fallen off over the years.

We glued both core and wedge samples onto wooden mounts and dried them for two weeks at room temperature. We sanded the samples with progressively finer sandpaper using belt and hand sanders, and then scanned each sample to create high-resolution digital images (1,200 dpi). We measured the ring width (mm) from bark to pith on all samples by WinDendro^™^ (Regent Instruments, 2008). If the pith was not present, measurements ended at the earliest ring formed. In accordance with other studies, the dendroecological approach has shown its effectiveness in the study of natural and anthropic disturbances in forest dynamics (Montoro Girona et al., 2016; Navarro et al., 2018; Montoro Girona et al., 2019).

We developed a master chronology based on the ring width of the cores taken from 76 healthy residual trees. The master chronology was used to identify any missing or false rings on the cores before we assigned a particular ring to a calendar year. We determined the year of tree death by cross-dating using COFECHA (Grissino-Mayer, 2001). However, because about 40% of wedges contained less than 30 years of rings, the master chronology was not applicable to all wedge samples. In these cases, we compared specific ring characteristics such as the contrast between early and late woods, width ratio between early and late wood per ring, or ring width on cores from 1960 to 2015. For example, tree rings were distinctively wider and had darker latewood in 1998 than in 1997, 1999, and 2000. Since the majority of sites experienced beetle mortality over a period of multiple years, the earliest tree death by MPB recorded at a site was considered as the year outbreak started at the site (**Table 1**; **Figure S2**).

**Table 1:** Number of study sites and residual *Pinus contorta* var. *latifolia* trees selected in each site from 2006 to 2010 during *Dendroctonus ponderosae* outbreak in Alberta (Canada).

	Year of attacks started in each site				
	2006	2007	2008	2010	Total
Number of sites	22	4	4	1	31
Number of trees sampled	144	38	22	6	210

We used a fixed sampling width (9 mm) of each annual ring on each increment core or wedge to quantify annual resin duct characteristics using ImageJ (Schneider et al., 2012): resin duct production (number of resin ducts in a 9mm wide sample area per year [no. yr^-1^]), total resin duct area (sum of resin duct area in a 9mm wide sampled area per year[mm^2^ yr^-1^]), resin duct size (mean size of resin ducts in a 9mm wide sampled area per year[mm^2^ yr^-1^]). We also standardized two resin duct characteristics to the sampled area as resin duct density (total number of resin ducts per year divided by the ring area (9mm * ring width) [no. mm^-2^ yr^-1^]) and relative resin duct area (percent area occupied by resin ducts per year within the ring area [% yr^-1^]) on each core or wedge.

We used ring width (mm yr^-1^) and basal area increment (BAI, [mm^2^ yr^-1^]) to represent tree radial growth. Working under the assumption that tree rings are concentric circles, the BAI was calculated using tree radii and ring width data. Tree radius was calculated by dividing tree DBH by two. Since we could not measure the DBH of beetle-killed trees, we only incorporated the BAI of residual trees in our data analyses.

### Data Analysis

We conducted separate analyses for each research question as described below. All statistical analyses were done in R Core Team (2018). We visually assessed the normality and homogeneity of variance of residuals for all models. Log transformations were applied when necessary. We then visually assessed the normality and homogeneity of variance of residuals of post-transformation models. A significance level (α) of 0.05 was used for all comparisons.

#### Can Radial Growth and Resin Duct Development Explain Why Residual Trees Were Not Killed During MPB Outbreak?

We pooled data from all residual trees together to test the differences in radial growth (ring width) and resin duct characteristics between residual and MPB-killed trees. We accepted the year of the earliest tree death caused by MPB as the year the outbreak started at each site.

We divided cores from residual trees into two sections: the pre-outbreak and the post-outbreak. Then, we further divided the pre-outbreak section into three overlapping periods: 3-, 5-, and 10-year pre-outbreak periods counting back from the year before the outbreak started at each site. Similarly, for MPB-killed trees, we selected three overlapping periods (3, 5, and 10 years) on wedges counting back from the last year when a complete tree ring (latewood) was formed (Cailleret et al., 2017). We first calculated the means of each of the five resin duct characteristics and the ring width for each growth period separately, and we then compared the means between residual and killed trees *via* linear mixed-effects models, using plots nested in sites as a random effect with R package *lme4* (Bates et al., 2015). When we found differences, we used lsmeans to conduct pairwise post-hoc tests using R package *lsmeans* (Lenth, 2016).

We used mixed effects logistic regression models to determine the probability of tree survival during MPB outbreak using R package *lme4*. The response variables for the models were the tree status (live or dead) and the explanatory variables included ring width and the five resin duct characteristics. All residual trees sampled were included in the analyses. We took the following five steps to select the best explanatory variables for model fitting.

In Step 1, we used principle component analysis (PCA) to visualize differences in the resin duct characteristics and ring width among the four categories of trees sampled using R package *vegan* (Oksanen et al., 2011). The two-dimension PCA plot illustrated the means of each of the explanatory variables of each growth period (3-, 5-, and 10-year) separately by vector arrows. Since the angles of the vector arrows indicated the correlation among variables, we classified vector arrows pointing in the same or similar directions as being in the same group (Ramette, 2007). This process separated the 18 explanatory variables into three distinct groups: (1) resin duct production and total resin duct area, (2) resin duct density and relative resin duct area, and (3) resin duct size and ring width (**Figure S3**).

In Step 2, we examined the correlations among all variables using a Pearson correlation test using an R package *Hmisc* (Harrell, 2008) (**Table S3**). P values lower than 0.05 indicated significant correlation between any two variables.

In Step 3, we collected the possible predictor combinations for the mixed effects logistic regression models using the variables that were separated into different groups according to the PCA plot (the results of Step 1 above) and that were not correlated to each other (the results of Step 2 above). This process yielded a total of 33 models (**Table 2**). Since ring width was strongly correlated with all resin duct characteristics, it was excluded from all predictor combinations at the beginning. However, excluding ring width from analyses may also lead us to an incorrect conclusion that it was not associated with tree survival. To solve this conundrum, instead of using the P value of the correlation test, we used correlation coefficients to determine the correlation between ring width and each resin duct characteristic. We only included ring width in the model if correlation coefficient value is less than 0.5 with any explanatory variable.

**Table 2:** The models predicting the survival probability of *Pinus contorta *var. *latifolia* trees using Akaike’s Information Criterion (AIC) and delta AIC (∆AIC), area under receiver operating curve (AUC), and internal validation results.

	Models	AIC	∆AIC	AUC	Killed trees correctly classified (%)	Residual trees correctly classified (%)
1	RDP10Y + RDS10Y	277.05	0	0.91	78.57	87.25
2	RDP5Y + RDS10Y	281.27	4.22	0.91	80.00	87.75
3	RDP5Y + RDS5Y	296.64	19.59	0.91	76.43	85.78
4	RDP10Y + RDS5Y	302.54	25.49	0.89	75.71	85.78
5	RDP5Y + RDS3Y + RW5Y	303.19	26.14	0.90	72.86	86.27
6	RDP3Y + RDS10Y	304.41	27.36	0.90	69.29	86.76
7	RDP10Y + RDS3Y + RW10Y	305.88	28.83	0.89	76.43	83.33
8	RDP5Y + RDS3Y + RRDA10Y	306.28	29.23	0.90	75.00	85.78
9	RDP3Y + RDS5Y	314.55	37.50	0.89	68.57	86.76
10	RDP3Y + RDS3Y + RRDA10Y	315.79	38.74	0.89	69.29	85.29
11	RDP3Y + RDS3Y	319.32	42.27	0.89	67.14	88.24
12	RDP3Y + RRDA10Y	358.94	81.89	0.85	61.43	86.76
13	RRDA3Y + RDS5Y + RW5Y	375.20	98.15	0.84	60.71	84.80
14	RDS3Y + RRDA5Y + RW10Y	380.65	103.6	0.83	57.86	83.82
15	RDA5Y + RDD5Y	381.93	104.88	0.83	56.43	83.33
16	RDS3Y + RRDA3Y + RW3Y	382.53	105.48	0.83	58.57	84.31
17	RDA3Y + RDD5Y	383.29	106.24	0.83	55.00	84.31
18	RDD5Y + RDA10Y	383.55	106.50	0.83	55.71	84.31
19	RDD3Y + RDA5Y	390.65	113.60	0.81	35.00	84.80
20	RDA5Y + RDD10Y	392.30	115.25	0.82	57.86	84.31
21	RDD3Y + RDA10Y	392.69	115.64	0.81	52.14	82.35
22	RDA3Y + RDD10Y	395.48	118.43	0.82	58.57	84.31
23	RDA10Y + RDD10Y	398.00	120.95	0.81	55.00	83.82
24	RDA3Y + RDD3Y	398.83	121.78	0.80	51.43	84.31
25	RDS3Y + RRDA10Y + RW10Y	408.23	131.18	0.81	57.14	82.84
26	RDA5Y + RRDA5Y	421.17	144.12	0.76	42.14	84.31
27	RRDA5Y + RDA10Y	422.22	145.17	0.77	39.29	82.35
28	RDA3Y + RRDA5Y	422.59	145.54	0.76	44.29	83.33
29	RRDA3Y + RDA5Y	423.10	146.05	0.75	42.86	84.8
30	RRDA3Y + RDA10Y	424.23	147.18	0.76	42.86	82.84
31	RDA5Y + RRDA10Y	434.08	157.03	0.75	40.00	84.31
32	RDA3Y + RRDA10Y	436.94	159.89	0.74	39.29	84.31
33	RDA10Y + RRDA10Y	437.88	160.83	0.75	36.43	83.33

RW, ring width (mm yr-1); RDP, resin duct production (no. yr^-1^); RDA, total resin duct area (mm^2^ yr^-1^); RDS, resin duct size (mm^2^ yr^-1^); RDD, resin duct density (no. mm^-2^ yr^-1^); RRDA, relative resin duct area (% yr^-1^). 3Y, 3-year pre-outbreak growth period; 5Y, 5-year pre-outbreak growth period; 10Y, 10-year pre-outbreak growth period.

In Step 4, we used the Akaike Information Criterion (AIC) to select the optimal set of variables in each model. The model with lower AIC was considered to have the optimal set of variables (Burnham and Anderson, 2002). We considered models with fewer variables as the optimal set of variables for the model.

In Step 5, we compared different models by calculating the AIC, delta AIC (∆AIC), and the area under receiver operating curves (AUC). The models with a lower AIC and higher AUC usually performed better than the models with a higher AIC or/and lower AUC (Hosmer and Lemeshow, 2000; Burnham and Anderson, 2002). The ∆AIC represents the difference in the AIC values between each model and the optimal model (Burnham and Anderson, 2002). We calculated the AUC using R package *pROC* (Robin et al., 2011).

#### Was the Growth and Resin Duct Development of Residual Trees Affected by MPB Outbreak?

To compare growth rate and resin duct characteristics before and after MPB outbreak, we divided cores into two sections as the pre-outbreak and the post-outbreak. The post-outbreak section was further divided into three overlapping periods: 3 years, 5 years, and the total (period from the first year after the outbreak until the last complete ring formation in 2015). Because the first year the outbreak started was different among sites, the total post-outbreak period was also different, ranging from 8 to 10 years.

We first calculated the means of all resin duct characteristics, ring width, and BAI for each post-outbreak period. We then compared these means with the means of the same variables calculated from the 10-year pre-outbreak period under the first research question above. These comparisons were made for every category of residual trees separately and using linear mixed-effects models, with trees nested in plots and plots nested in sites as a random effect. We used DBH as a covariant to account for the influence of tree size. When we found differences, we used lsmeans to conduct pairwise post-hoc tests.

#### Can Growth and Resin Duct Development Explain Differences in the Health Conditions of Residual Trees?

We selected four overlapping periods on each increment core: 3-, 5-, 10-, and 20-year, counting back from the last complete ring formation in 2015. We then calculated the mean of each resin duct characteristic, ring width, and BAI for each period and compared their means among residual tree categories using linear mixed-effects models, with plots nested in sites as a random effect. We used tree DBH as a covariant to account for the influence of different tree sizes in these models. When we found differences, we used lsmeans to conduct pairwise post-hoc tests.

## Results

### Can Radial Growth and Resin Duct Development Explain Why Residual Trees Were Not Killed During MPB Outbreak?

**Figure 1:**
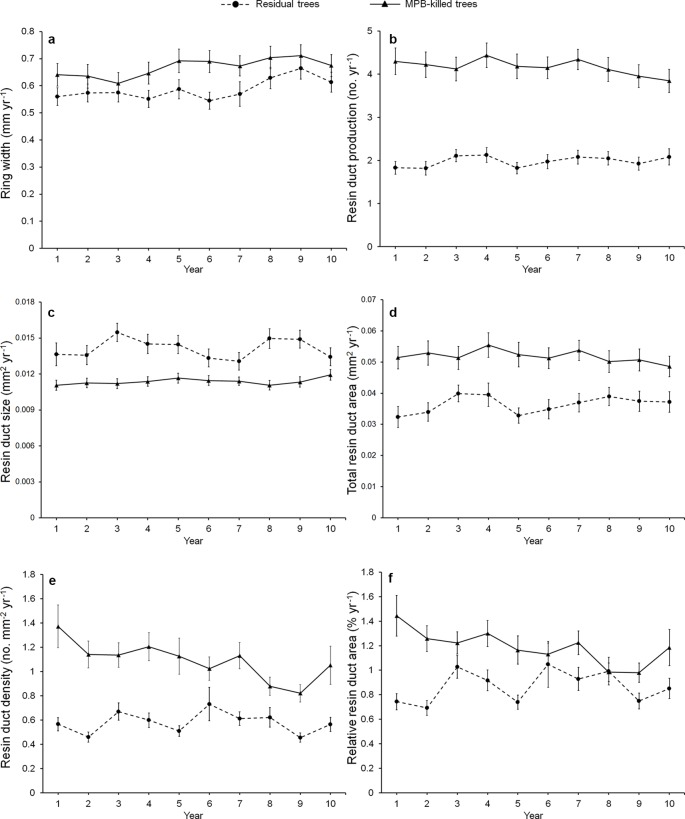
Annual variation in the mean of ring width **(a)**, resin duct production **(b)**, resin duct size **(c)**, total resin duct area **(d)**, resin duct density **(e)**, and relative resin duct area **(f)** of residual and killed *Pinus contorta *var. *latifolia* trees in the 10 years before mountain pine beetle (MPB, *Dendroctonus ponderosae*) outbreak. We did not use calendar years because the starting year of MPB outbreak varied among sites and the year of trees killed varied among trees.

Mean ring width was similar between beetle-killed and residual trees for all three pre-outbreak (3-, 5-, and 10-year) periods (**Figure 1a**), whereas all resin duct characteristics showed significant differences in all three pre-outbreak periods (**Table 3**). The mean resin duct production of killed trees was 119–123% greater than those residual trees (**Figure 1b**). Likewise, killed trees had a 46–53% higher total resin duct area, 105–131% greater resin duct density, and 57–78% higher relative resin duct area than residual trees (**Figures 1d–f**). However, residual trees had 14–24% larger resin ducts than killed trees (**Figure 1c**).

**Table 3:** Comparisons of mean resin duct characteristics of killed and residual *Pinus contorta* var. *latifolia* trees over 3, 5, and 10 years before *Dendroctonus ponderosae* outbreak in Alberta. Medians and 25% and 75% quartile of raw data are listed in the table.

Variables	Median (25%, 75% quartile)		F-value	P
	Residual trees	Killed trees		
***Resin duct production (no. yr*** ***^-1^*** ***)***				
3-year	1.667 (1.000, 2.333)	3.667 (2.667, 5.417)	126.08	<0.001
5-year	1.600 (1.000, 2.600)	3.800 (2.800, 5.600)	199.22	<0.001
10-year	1.600 (1.000, 2.600)	3.850 (2.875, 5.250)	201.54	<0.001
***Resin duct density (no. mm*** ***^-2^*** *** yr*** ***^-1^*** ***)***				
3-year	0.439 (0.222, 0.673)	0.863 (0.575, 1.431)	91.847	<0.001
5-year	0.441 (0.237, 0.703)	0.787 (0.599, 1.498)	104.34	<0.001
10-year	0.471 (0.233, 0.796)	0.865 (0.577, 1.330)	88.169	<0.001
***Relative resin duct area (% yr*** ***^-1^*** ***)***				
3-year	0.663 (0.353, 1.037)	1.054 (0.738, 1.542)	51.299	<0.001
5-year	0.662 (0.416, 1.048)	0.969 (0.773, 1.528)	59.195	<0.001
10-year	0.702 (0.369, 1.106)	1.020 (0.756, 1.469)	45.124	<0.001
***Total resin duct area (mm*** ***^2^*** *** yr*** ***^-1^*** ***)***				
3-year	0.030 (0.016, 0.044)	0.046 (0.030, 0.069)	33.49	<0.001
5-year	0.028 (0.017, 0.046)	0.047 (0.030, 0.067)	23.682	<0.001
10-year	0.029 (0.017, 0.045)	0.047 (0.033, 0.067)	45.449	<0.001
***Resin duct size (mm*** ***^2^*** *** yr*** ***^-1^*** ***)***				
3-year	0.014 (0.009, 0.019)	0.012 (0.009, 0.013)	12.248	<0.001
5-year	0.014 (0.009, 0.019)	0.011 (0.009, 0.013)	20.578	<0.001
10-year	0.014 (0.010, 0.018)	0.012 (0.010, 0.013)	25.086	<0.001

Resin duct characteristics were log-transformed to stabilize the normality and homogeneity of variance of model residuals.

**Figure 2:**
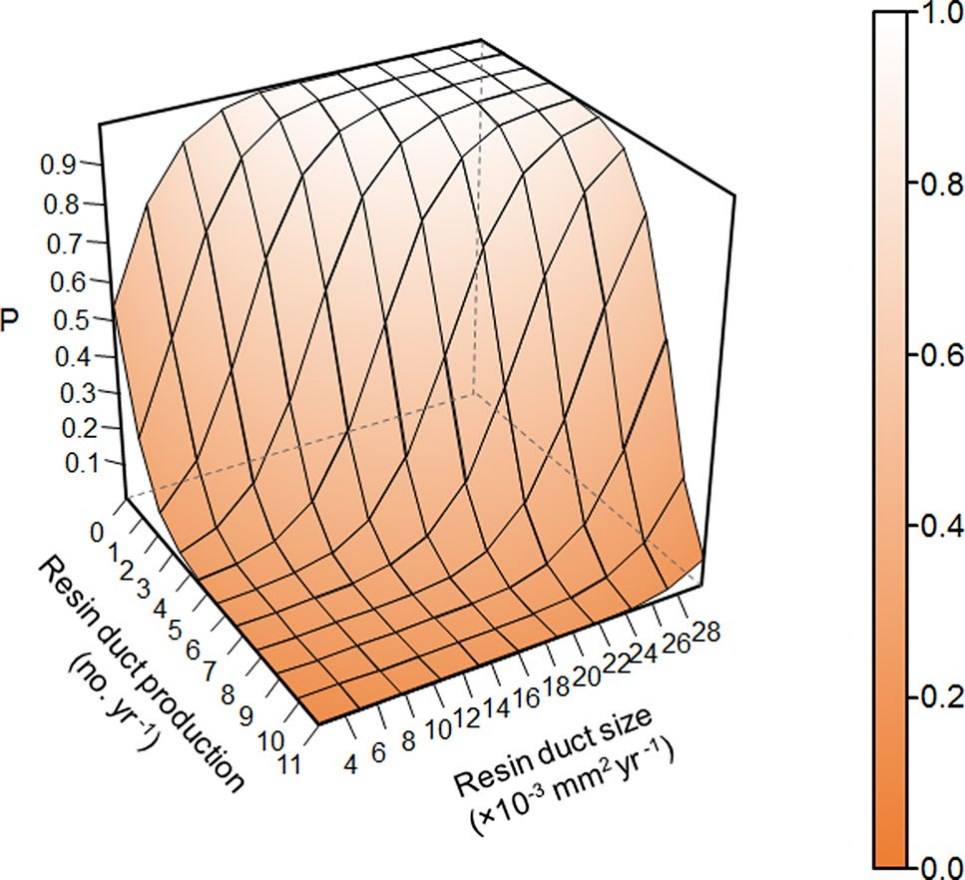
Prediction of survival probability of *Pinus contorta* var. *latifolia* trees under *Dendroctonus ponderosae* attacks from the best model in **Table 2** based on 10-year mean resin duct production and resin duct size of *P. contorta* var. *latifolia* trees in post-outbreak stands excluding random effects. P represents the survival probability of *P. contorta* var. *latifolia* trees under *D. ponderosae* attacks.

Overall, resin duct characteristics were more important for estimating the survival probability of pine trees than ring width in models. Models classified residual trees as better than killed trees (82–88% vs. 36–80% respectively). Models including both resin duct production and resin duct size had a lower AIC (< 320) and higher AUC (≥ 0.89) and performed better in classifying killed and residual trees (67-80% vs. 83-88% respectively), compared to the models that included only one of the two resin duct characteristics or none (AIC > 350, AUC ≤ 0.85) (**Table 2**). The best predictive model included the 10-year mean resin duct production and the 10-year mean resin duct size (**Table 2**; **Figure 2**) with the lowest AIC (277.05) and the highest AUC (0.91).

### Was the Growth and Resin Duct Development of Residual Trees Affected by MPB Outbreak?

**Figure 3:**
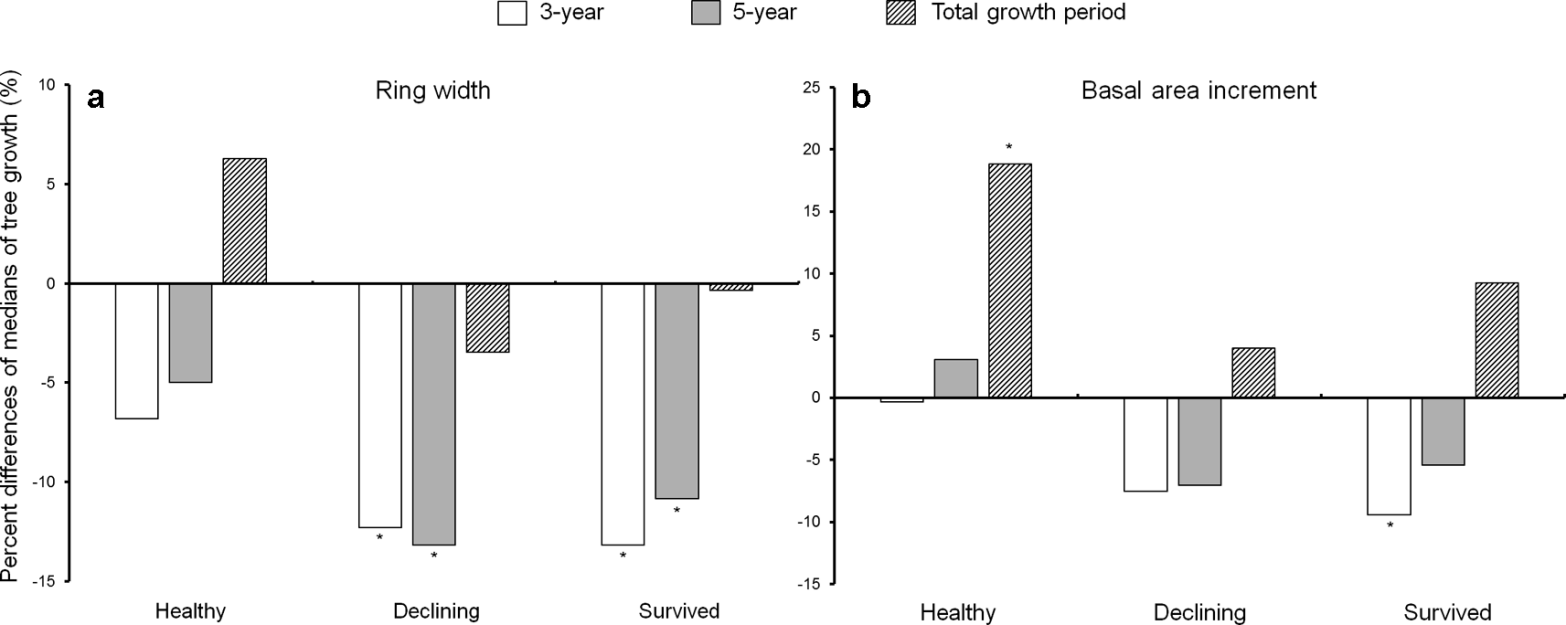
Percent difference of medians of ring width **(a)** and basal area increment **(b)** between the 10-year growth period before *Dendroctonus ponderosae* outbreak and each of the three growth periods after outbreak (3-, 5-year and total growth period) in each of the three categories of residual *Pinus contorta* var. *latifolia* (healthy, declining, and survived) trees. Significant differences between growth before and after outbreak (α = 0.05) were indicated by asterisks (*). Both ring width and basal area increment were log-transformed to stabilize the normality and homogeneity of variance of model residuals.

Both declining and survived trees showed reduced ring width during the first 5 years after the outbreak as compared to before the outbreak (**Figure 3**). While the ring width of healthy trees did not change between the pre- and post-outbreak growth periods (**Figure 3a**), healthy trees had increased BAI after outbreak. The BAI of declining trees did not vary due to the outbreak, while survived trees showed lower BAI in the first 3 years after the outbreak (**Figure 3b**). As a covariant, DBH was not significant between pre- and post-outbreak periods in any of the models (P > 0.05).

**Figure 4:**
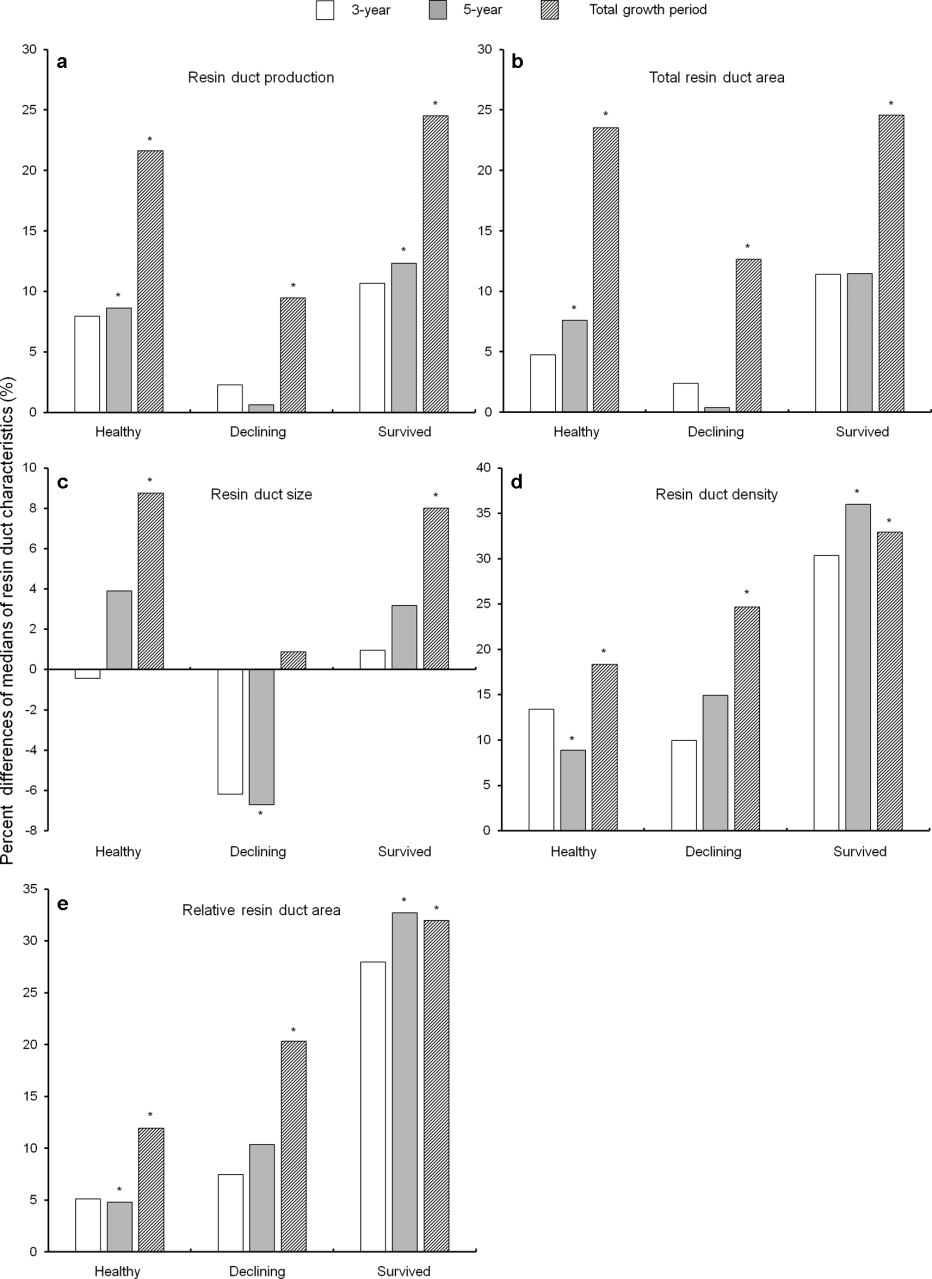
Percent differences of medians of resin duct production **(a)**, total resin duct area **(b)**, resin duct size **(c)**, resin duct density **(d)**, and relative resin duct area **(e)** between the 10-year growth period before *Dendroctonus ponderosae* outbreak and each of the three growth periods after outbreak (3-, 5- and total growth period) in three categories of residual *Pinus contorta *var. *latifolia* (healthy, declining, and survived) trees. Significant differences between resin duct characteristics before and after outbreaks (α = 0.05) were indicated by asterisks (*). All resin duct characteristics were log-transformed to stabilize the normality and homogeneity of the variance of model residuals.

In general, almost all resin duct characteristics in all residual trees had a higher value in the post-outbreak growth periods as compared to the pre-outbreak growth period (**Figure 4**). After the outbreak, healthy and survived trees had higher resin duct production in the 5-year and total growth periods, while declining trees showed higher resin duct production only in the total growth period (**Figure 4a**). Total resin duct area in healthy trees increased in the 5-year and total growth periods, while both survived and declining trees had higher total resin duct area in the total growth period (**Figure 4b**). The size of resin ducts increased in both healthy and survived trees in the total growth period. Declining trees had smaller resin ducts in the 5-year growth period compared to the pre-outbreak growth period, but the resin duct sizes in the total growth period were similar between the pre- and post-outbreak periods (**Figure 4c**). Both healthy and survived trees showed a significantly higher resin duct density and larger relative resin duct area in both the 5-year and the total growth periods. Declining trees had a higher resin duct density and larger relative resin duct area only in the total post-outbreak growth period (**Figures 4d, e**).

As a covariant, DBH was significant in models that compared resin duct production of healthy trees between the 3-year post-outbreak and the pre-outbreak periods, total resin duct area of healthy trees between the 3-year post-outbreak and the pre-outbreak periods, and total resin duct area of healthy trees between 5-year post-outbreak and pre-outbreak periods.

### Can Growth and Resin Duct Development Explain Differences in the Health Conditions of Residual Trees?

**Figure 5:**
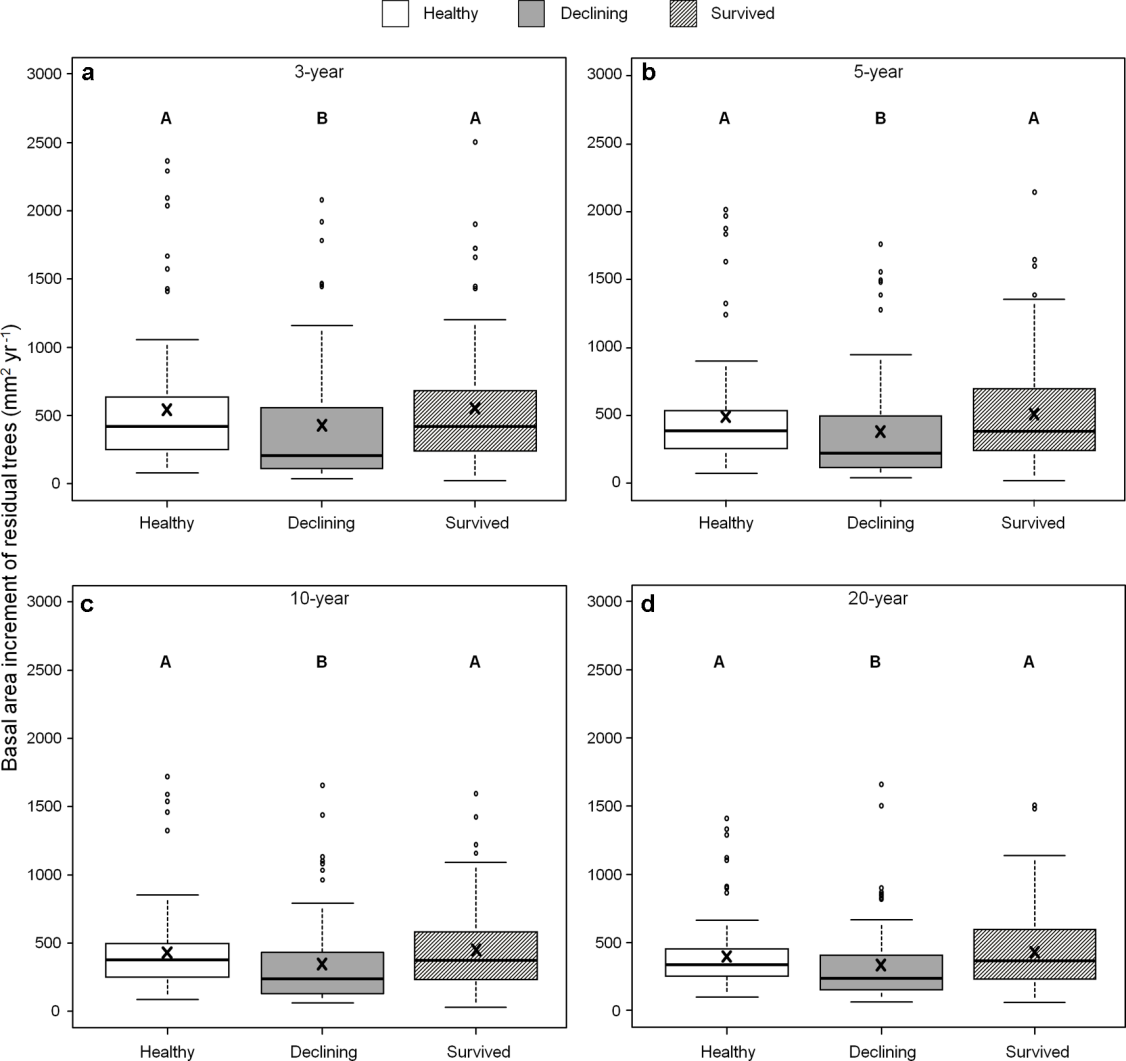
Basal area increments of three categories (healthy, declining, survived) of residual *Pinus contorta* var. *latifolia* trees in the four most recent growth periods (3 **(a)**, 5 **(b)**, 10 **(c)**, and 20 years **(d)**). Significant differences among three tree categories were indicated by different letters based on the results of linear mixed-effects models (α = 0.05). In each box plot, the thick line is the median, × mark indicates the mean, the box represents the 1st and 3rd quartiles, whiskers are Tukey’s 1.5 interquartile range, and the open circles indicate outliers. Basal area increment was log-transformed to stabilize the normality and homogeneity of variance of model residuals.

We found no differences in ring width among the three categories of residual trees for the recent 3-, 5-, 10- and 20-year growth periods. By contrast, BAI varied among residual trees (**Figure 5**) and both healthy and survived trees had consistently larger BAI than declining trees in all four periods (healthy: 43–108%; survived: 54–106%). As a covariant, DBH was significant in models that compared ring width among the categories of residual trees (P < 0.05).

**Figure 6:**
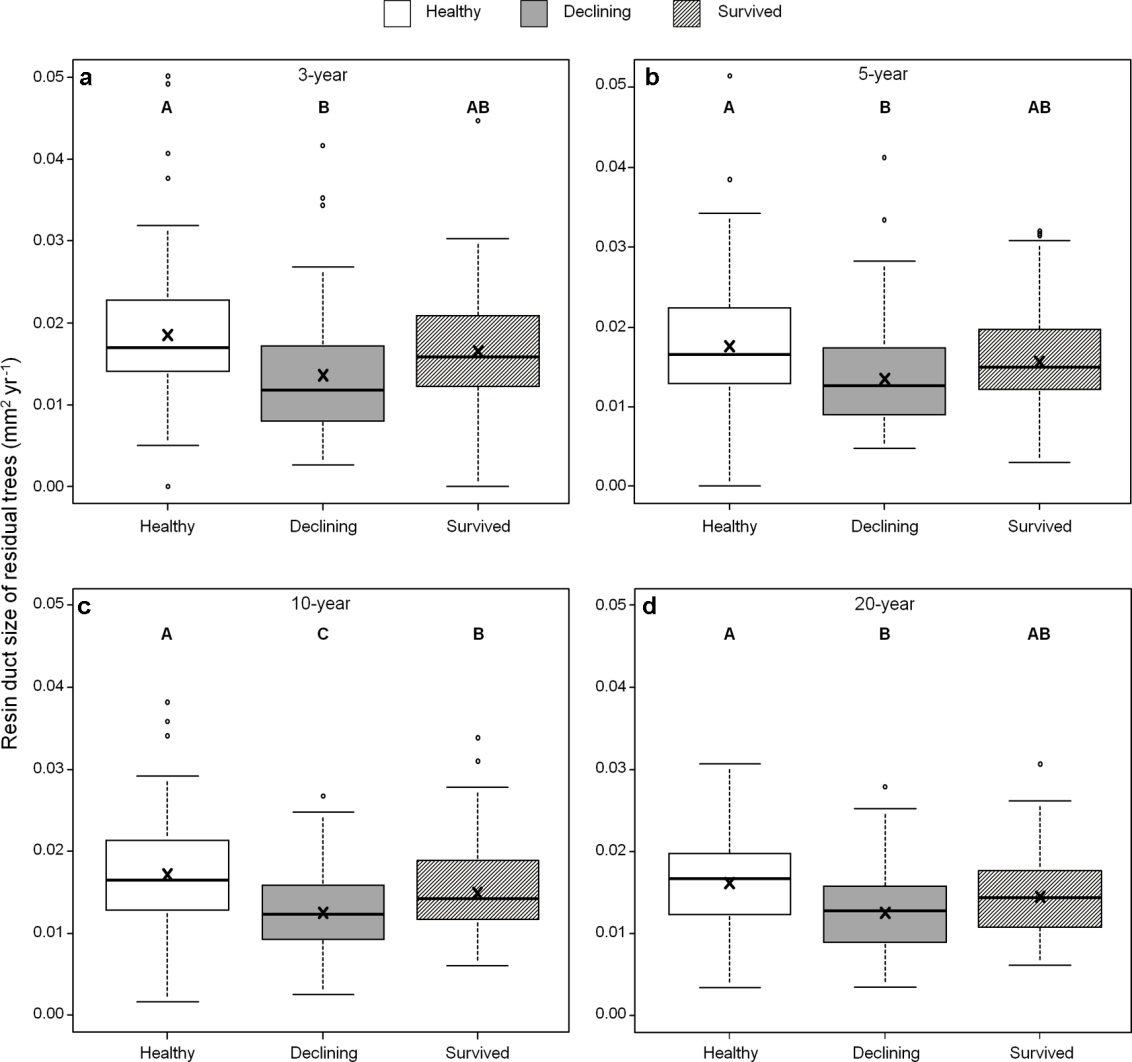
Resin duct size of three categories (healthy, declining, survived) of residual *Pinus contorta *var. *latifolia* trees in four most recent growth periods (3 **(a)**, 5 **(b)**, 10 **(c)**, 20 years **(d)**). Significant differences among three tree categories were indicated by different letters based on the results of linear mixed-effects models (α = 0.05). In each box plot, the thick line is the median, × mark indicates the mean, the box represents the 1st and 3rd quartiles, whiskers are Tukey’s 1.5 interquartile range, and the open circles indicate outliers Resin duct size was log-transformed to stabilize the normality and homogeneity of variance of model residuals.

**Figure 7:**
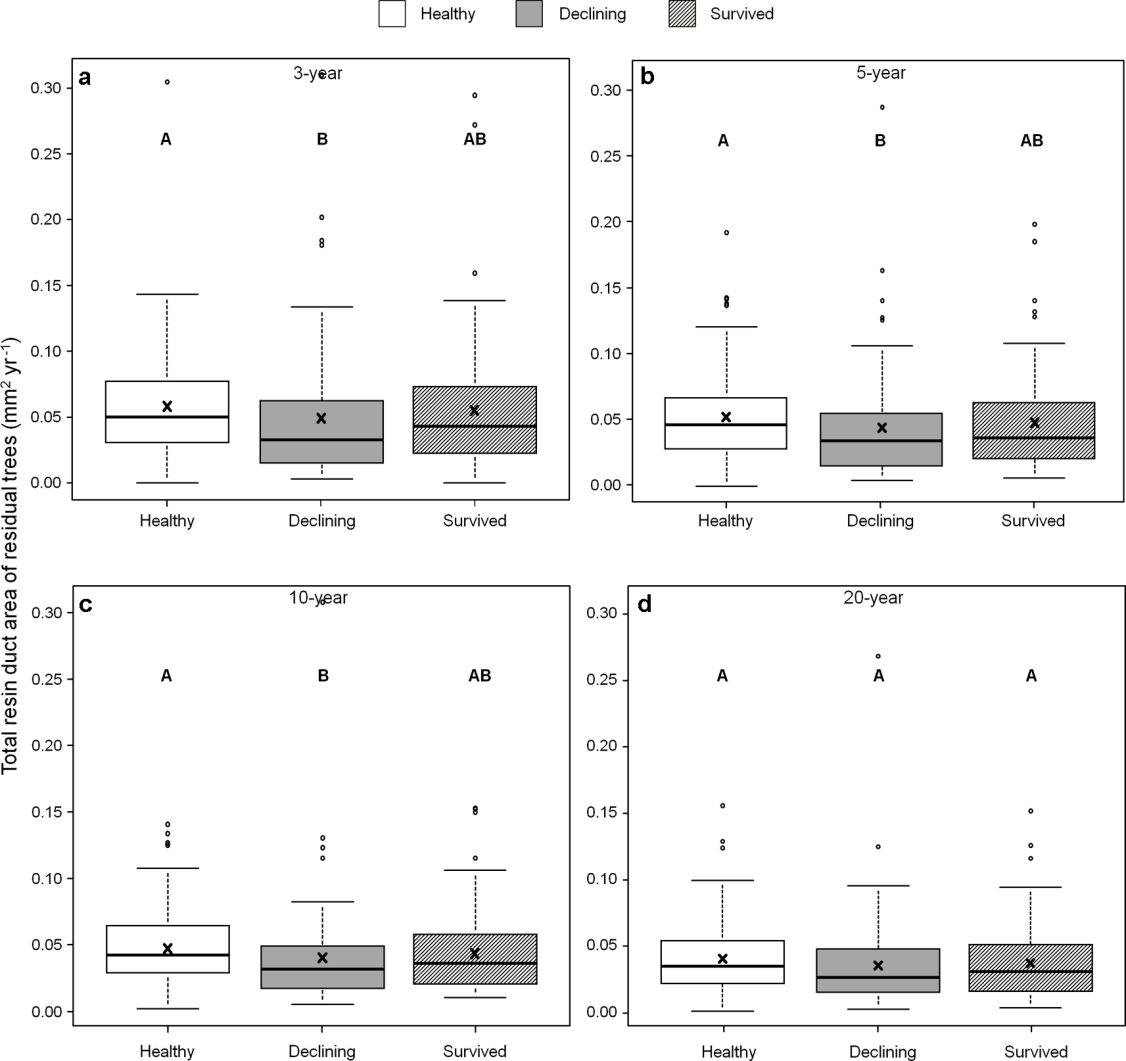
Total resin duct area of three categories (healthy, declining, and survived) of residual *Pinus contorta* var. *latifolia* trees in four most recent growth periods (3 **(a)**, 5 **(b)**, 10 **(c)**, and 20 years **(d)**). Significant differences among three tree categories were indicated by different letters based on the results of linear mixed-effects models (α = 0.05). In each box plot, the thick line is the median, × mark indicates the mean, the box represents the 1st and 3rd quartiles, whiskers are Tukey’s 1.5 interquartile range, and the open circles indicate outliers. Total resin duct area was log-transformed to stabilize the normality and homogeneity of variance of model residuals.

Among all five resin duct characteristics, only resin duct size (**Figure 6**) and total resin duct area (**Figure 7**) varied among residual trees. Healthy trees had 28–44% larger resin duct sizes compared to declining trees in all four growth periods. The resin duct size of healthy trees was 16% larger than survived trees in the 10-year period, while survived trees had 15% larger resin duct size than declining trees in the same period. For other time periods, the size of the resin ducts of survived trees was not different from the other two residual tree categories. Healthy trees had a 36–44% larger total resin duct area than declining trees in the 3-, 5- and 10-year periods, while survived trees did not show any differences in total resin duct area in any periods compared to the other two categories of residual trees. All residual trees had similar total resin duct area in the last 20-year growth period. As a covariant, DBH was significant in all models that compared resin duct production in the 10- and 20-year periods (P > 0.05).

## Discussion

Through this study, we showed that resin duct characteristics can be critical components of pine survival before and after MPB outbreaks. Overall, beetle-killed trees had smaller resin ducts than residual trees and residual trees continued to have larger resin ducts after MPB outbreak. By increasing the volume of resin flow, larger resin ducts likely substantially reduced the probability of successful beetle colonization on residual trees by providing sticky physical barriers, sealing beetle entry wounds, and releasing toxic compounds (Franceschi et al., 2005; Erbilgin et al., 2017; Erbilgin, 2019; Mason et al., 2019). Thus, these results are in agreement with earlier studies emphasizing the importance of anatomic defenses in the survival of conifers against tree-killing bark beetle species in North America (Kane and Kolb, 2010; Ferrenberg et al., 2014; Gaylord et al., 2015; Hood and Sala, 2015; Mason et al., 2019).

Among the anatomical characteristics measured, the number of resin ducts did not seem to be as important as their size; lodgepole pine trees with relatively smaller but more resin ducts were killed during MPB outbreak, and trees with larger but fewer resin ducts had a higher probability of survival during outbreak, suggesting a possible trade-off between size and number of resin ducts in pine trees (Herms and Mattson, 1992). In addition, residual pine trees with larger resin ducts were healthy compared to the declining trees, which had relatively smaller resin ducts. These results indicated that resin duct size likely played a critical role in pine survival during MPB outbreak in the current study. We provided four possible explanations to support our results.

First, larger resin ducts likely result in the storage and biosynthesis of a higher volume of resin, thereby increasing resin accumulation within the tree (Hood and Sala, 2015). Consequently, such trees could form a stronger constitutive defense line against bark beetles. Second, larger resin ducts could rapidly deploy a much higher amount of resin flow to the beetle attack points, which would increase the likelihood of the entrapment of beetles at the host entrance during initial host colonization (Schopmeyer et al., 1954; Hood and Sala, 2015; Cale et al., 2017; Erbilgin et al., 2017). Using the Poiseuille’s Law, one unit increasing in the radius of resin ducts can result in resin flow increasing by a fourth power. Third, larger resin ducts can transport higher viscosity resin within trees (Tyree and Zimmermann, 2002). With higher viscosity, resin can carry higher amounts of terpenes and be more effective during beetle attack (Franceschi et al., 2005). Finally, the resin duct size of residual trees in the healthy and survived categories either remained the same or increased after MPB outbreak, suggesting possible effects of tree genetics and genetics–environment interaction on resin duct size. While some resin duct characteristics (density and production) are influenced by tree genetics in some pine species (Moreira et al., 2015; Westbrook et al., 2015), the heritability of resin duct size remains unclear in lodgepole pine and requires further investigations. Nevertheless, the survival of pine trees with larger resin ducts suggests that bark beetle outbreaks likely drive selection for better-defended lodgepole pine phenotypes.

Our results emphasized the importance of resin duct size over the number of resin ducts. These results are partially in disagreement with former studies that reported a higher number of resin ducts in resistant trees (Kane and Kolb, 2010; Ferrenberg et al., 2014). Above, we discussed the advantages trees can have during high-density bark beetle attacks if they have larger resin ducts; however, having fewer large ducts may also limit their ability to defend the entire tree stem if there are multiple points of attack along the stem. In contrast, trees with many smaller resin ducts may have the ability to defend trees at many points of attack along stem, but they will probably fail to develop a quick and lethal induced defense response against bark beetles. Historically insect-host plant interactions may also affect the development of anatomical defense. Future studies should investigate this possible trade-off between efficient resin flow (larger resin ducts) on one hand and reduced defense coverage (fewer resin ducts) on the other hand.

We consistently found enhanced anatomical defense structures in residual pine trees during post-outbreak as compared to pre-outbreak periods. This is the first report of enhanced anatomical defenses in pines after bark beetle outbreaks. Two possible post-outbreak stand conditions may explain these results. First, these stands may not provide optimal conditions for tree growth following MPB outbreaks (Cigan et al., 2015; Karst et al., 2015; Pec et al., 2017). In fact, the reduced growth in residual trees right after an outbreak in the current study may reflect less optimal growing conditions in these stands and a possible trade-off between anatomical defense and tree growth. This is expected as the growth-differentiation balance hypothesis predicts that plants favor defense over growth when resources are limited (Herms and Mattson, 1992). Second, it has been widely documented that plants, including lodgepole pine, under attack from herbivory insects can release volatile organic compounds that can alarm neighboring trees (Baldwin and Schultz, 1983; Engelberth et al., 2004; Hussain et al., 2019), which might have led to the production of additional resin ducts in the healthy trees. This explanation warrants further studies in the field.

Overall ring width was similar between beetle-killed and residual trees and was not associated with pine survival in the best predictive models. However, ring width was correlated with tree size, suggesting that, with the same ring width, larger trees can have more annual BAI compared to smaller trees. Therefore, DBH should have been used as a covariant to account for the influence of tree size in our models. However, since we could not accurately measure the DBH of all beetle-killed trees, this created some potential problems for data analysis and interpretation. To resolve this issue, we used the DBH of beetle-killed trees with intact bark for further comparison with the DBH of residual pine trees. These results showed that beetle killed trees had larger DBH than residual trees (**Figure S4**), indicating that dead trees likely grew faster than residual trees before MPB outbreak, in agreement with the results of an earlier study (de la Mata et al., 2017; Cooper et al., 2018). The role of tree growth as an indicator of pine resistance or susceptibility to bark beetles is not clear and may reflect differences in stand conditions, such as percent tree mortality, forest structure and composition, competition index, spatial position, and climate (Campbell et al., 2007; Kane and Kolb, 2010; Hawkins et al., 2013; Ferrenberg et al., 2014; Montoro Girona et al., 2016; Pec et al., 2015; Pec et al., 2017). For example, in our study, residual trees showed increasing growth rates five years after MPB outbreak, while earlier studies reported accelerated radial growth as early as three years (Murphy et al., 1999; Hawkins et al., 2013; Dhar et al., 2016). For future research, we recommend exploring the growth response using individual non-linear models to get a better resolution of tree growth after insect outbreaks along with increasing sample size to better understand the variability in the tree growth responses (Montoro Girona et al., 2017).

In Alberta, MPB outbreaks happened unexpectedly as a large number of beetles dispersed from British Columbia and landed in the forests in the west of Alberta. In contrast to the historical range of MPB, Alberta did not experience the regular population cycles of beetles (endemic, incipient-epidemic, and epidemic) (Safranyik et al., 2010). Although the lodgepole pine trees we sampled likely experienced MPB attacks during a short period of time from 2006 to 2010 (**Figure S2**), there was also tree mortality beyond this period. These conditions potentially introduced complexity to our study. For example, we used the earliest tree death caused by MPB at each site as the year the outbreak started and then divided the growth series into pre- and post-outbreak periods; we may have included tree ring data from trees that were not attacked in the earliest year as “outbreak affected”.

In conclusion, we showed that anatomical defense structures can be critical components of lodgepole pine survival during MPB outbreaks. Furthermore, the same defense structures have likely continued playing major roles in defending residual trees against biotic attacks in post-outbreak stands. Among anatomical defense structures investigated, resin duct size appears to be an important indicator of tree resistance to bark beetles. However, the literature on the resin duct characteristics of conifers shows variations, but underlying factors for such differences are not clear and require further investigations. It is not clear whether resin duct size is heritable, but, if so, we expect that the next generation of lodgepole pine forests in western Alberta would be more resistant to future MPB attacks. Considering the climate change enhanced bark beetle outbreaks in North America (Raffa et al., 2008; Bentz et al., 2010), we suggest retaining these residual trees in stands, which could help to establish the future sustainable lodgepole pine forests in Alberta. For example, these residual trees can be used for seed tree selection to be used in tree improvement programs.

## Data Availability Statement

The datasets generated for this study are available on request to the corresponding author.

## Author Contributions

SZ and NE conceived and designed the experiments. SZ performed the experiments. SZ and NE performed statistical analysis and generated tables and figures. SZ wrote the manuscript. NE contributed to editing.

## Funding

Funding for this research was provided by fRI Research, Alberta Agriculture and Forestry, Alberta Innovates-BioSolutions, Forest Resource Improvement Association of Alberta, and NSERC–Discovery Award to NE. 

## Conflict of Interest

The authors declare that the research was conducted in the absence of any commercial or financial relationships that could be construed as a potential conflict of interest.
