# Supplementary material for: Larger Resin Ducts Are Linked to the Survival of Lodgepole Pine Trees During Mountain Pine Beetle Outbreak
Source: Front Plant Sci. 2019 Nov 26;10:1459. doi: 10.3389/fpls.2019.01459 (PMC6888816; doi:10.3389/fpls.2019.01459)
Supplement: Supplementary file 1 [file Table_1.pdf]

Supplementary Material

Table S1. Stand age, density and percent basal area of each species of mature overstory trees presented in selected sites in western Alberta, Canada.

Site No.	Stand Age	*Pinus contorta* var. *latifolia*		*Picea glauca*		*Populus tremuloides*		*Abies balsamea*		*Betula papyrifera*		*Picea mariana*		*Populus balsamifera*	
		Density	Basal area (%)	Density	Basal area (%)	Density	Basal area (%)	Density	Basal area (%)	Density	Basal area (%)	Density	Basal area (%)	Density	Basal area (%)
1	103	65	99.10%							1	0.90				
2	113	43	78.91%	7	14.47	2	4.17	3	2.44						
3	102	19	86.01%	5	13.99										
4	101	19	64.03%	11	14.84	4	21.13								
5	75	29	79.80%	9	13.47	3	6.73								
6	80	42	75.45%			7	21.56			3	2.99				
7	74	43	68.71%	9	10.23	17	18.44	3	2.62						
8	49	43	64.24%	2	1.98	25	32.58	1	1.21						
9	101	24	79.59%	6	20.41										
10	59	62	100.00%												
11	64	53	98.53%	1	1.47										
12	40	15	62.46%	14	36.04	1	1.49								
13	110	15	97.94%	1	2.06										
14	88	22	61.26%	25	38.07	1	0.66								
15	57	74	93.21%	4	4.99	1	1.04					1	0.77		
16	66	45	67.44%	5	5.64	11	21.97	1	1.18	1	1.36			1	2.41
17	54	50	93.92%			1	6.08								
18	97	30	55.20%	11	24.87	5	18.94								
19	60	43	93.93%	3	6.07										
20	125	66	68.16%	30	22.18	9	9.66								
21	110	29	63.31%	2	3.25	19	33.44								

22	62	42	98.61%							1	1.39
23	130	76	91.58%	7	7.64	1	0.78				
24	64	51	76.88%	5	5.59	8	15.36			2	1.50
25	109	56	86.76%	11	13.24						
26	45	14	70.03%	13	29.97						
27	123	61	68.79%	31	25.33	6	5.88				
28	100	16	55.32%	6	25.32	7	19.37				
29	100	35	66.29%	33	33.71						
30	NA	15	84.86%	4	15.14						
31	57	45	93.34%	3	6.66						

Stand age was determined by increment cores taken from *Pinus contorta* var. *latifolia* trees. Stand age of site No.30 was missing because increment cores taken in site No.30 were either damaged or not sampled to tree pith. Density of each species of trees includes only mature overstory trees having diameter at breast height over 15cm. Basal area (%) was calculated based on the basal area of each species of trees having diameter at breast height over 15cm and the total basal area of all trees having diameter at breast height over 15cm.

Table S2. Percent mortality of *Pinus contorta* var. *latifolia* trees caused by *Dendroctonus ponderosae* in selected sites in western Alberta, Canada.

No. Site	No of killed pine trees (ha)	Percent Mortality (%)	Diameter of Trees at Breast Height (cm) Mean (SE)			
			Killed	Healthy	Declining	Survived
1	600	82.76	28.74 (0.70)	NA	22.90 (1.92)	26.33 (1.03)
2	287.5	76.67	30.27 (0.89)	29.70 (0.00)	27.40 (1.46)	23.80 (0.07)
3	162.5	68.42	24.30 (2.26)	38.10 (2.76)	NA	23.05 (1.32)
4	162.5	68.42	33.40 (2.33)	43.60 (0.00)	NA	30.22 (2.60)
5	212.5	65.38	22.45 (0.89)	23.68 (1.54)	NA	NA
6	312.5	60.98	31.96 (1.02)	21.70 (0.00)	23.78 (1.25)	27.59 (1.86)
7	312.5	60.98	23.55 (0.55)	19.62 (1.19)	20.70 (1.72)	20.00 (0.00)
8	312.5	59.52	21.87 (0.74)	20.26 (0.44)	19.46 (0.84)	17.90 (1.91)
9	162.5	54.17	26.60 (1.33)	NA	28.40 (0.00)	26.60 (1.21)
10	362.5	50.88	22.48 (0.69)	19.45 (1.16)	19.24 (0.63)	18.75 (0.91)
11	300	50.00	19.20 (0.63)	18.70 (0.00)	17.50 (0.76)	17.91 (0.76)
12	87.5	46.67	24.63 (2.62)	22.30 (0.00)	NA	24.93 (1.59)
13	87.5	46.67	26.71 (1.97)	NA	NA	25.83 (1.23)
14	125	45.45	36.18 (1.82)	26.15 (0.81)	23.41 (1.47)	35.93 (3.40)
15	400	43.24	20.66 (0.73)	20.02 (0.67)	18.97 (0.50)	NA
16	237.5	43.18	20.19 (0.88)	18.31 (0.89)	17.65 (0.43)	19.15 (1.52)
17	250	41.67	20.52 (0.74)	16.25 (0.81)	19.15 (0.89)	18.65 (0.50)
18	150	41.38	30.54 (1.53)	32.00 (0.00)	26.80 (4.74)	25.94 (1.29)
19	200	39.02	17.79 (0.52)	18.27 (0.84)	17.20 (0.46)	15.90 (0.00)
20	175	25.93	22.85 (1.23)	21.49 (0.52)	21.68 (1.12)	22.41 (1.32)
21	75	24.00	34.93 (2.62)	20.30 (1.91)	32.44 (3.08)	25.07 (1.08)
22	100	19.05	19.81 (0.90)	19.21 (0.64)	19.37 (0.62)	20.50 (1.56)
23	137.5	16.67	23.62 (1.78)	22.55 (0.66)	20.04 (0.53)	25.61 (1.52)
24	100	16.33	25.51 (1.00)	23.30 (1.35)	21.19 (0.53)	NA
25	87.5	14.29	27.11 (1.80)	23.69 (0.92)	23.32 (0.88)	25.50 (1.56)
26	25	14.29	40.90 (2.26)	34.60 (0.00)	18.00 (0.00)	27.20 (4.31)
27	100	13.79	22.33 (1.10)	21.26 (1.77)	20.11 (0.73)	21.67 (0.69)
28	25	12.50	32.05 (6.54)	34.25 (4.12)	42.20 (0.00)	36.38 (1.94)
29	50	11.76	30.38 (1.73)	28.07 (2.76)	24.42 (0.87)	26.27 (1.91)
30	12.5	6.67	16.50 (0.00)	20.03 (1.49)	18.05 (1.26)	24.65 (1.59)
31	12.5	2.22	16.40 (0.00)	17.20 (0.43)	17.78 (0.57)	18.43 (1.44)

Density of beetle-killed trees (ha) was standardized by the density of killed pine trees in each site. Percent mortality of trees was calculated based on the density of dead pines and the density of all (dead or live) pine trees in each site. Healthy pine trees do not show any symptoms of

pathogen or insect attacks; declining pine trees show presence of insect, other than *D. ponderosae*, and pathogen attacks, along with dying branches, bark lesions, sparse crown, yellow-red needles; survived pine trees show symptoms of unsuccessful *D. ponderosae* attacks but appeared healthy.

Table S3 Correlation among growth and resin duct characteristics of residual *Pinus contorta* var. *latifolia* trees over 3, 5, and 10 years before *Dendroctonus ponderosae* outbreak and trees killed by *D. ponderosae* over 3, 5, and 10 years before tree death in post-outbreak stands in Alberta.

	3-year measurement						***5-year measurement***						***10-year measurement***				
	RW	RDP	RDA	RDS	RDD	RRDA	RW	RDP	RDA	RDS	RDD	RRDA	RW	RDP	RDA	RDS	RDD
3-year measurement																	
RDP	0.40 ***																
RDA	0.55 ***	0.84 ***															
RDS	0.34 ***	***0.09***	0.39 ***														
RDD	-0.38 ***	0.29 ***	***0.05***	-0.20 ***													
RRDA	-0.38 ***	0.26 ***	0.12 *	***-0.02***	0.91 ***												
5-year measurement																	
RW	0.99 ***	0.43 ***	0.57 ***	0.34 ***	-0.36 ***	-0.36 ***											
RDP	0.43 ***	0.93 ***	0.79 ***	***0.06***	0.23 **	0.20 ***	0.46 ***										
RDA	0.57 ***	0.79 ***	0.94 ***	0.35 ***	***0.00***	***0.05***	0.60 ***	0.85 ***									
RDS	0.40 ***	***0.06***	0.38 ***	0.90 ***	-0.27 ***	***-0.10***	0.40 ***	***0.06***	0.39 ***								
RDD	-0.40 ***	0.19 ***	***-0.02***	-0.23 ***	0.92 ***	0.81 ***	-0.40 ***	0.24 ***	***0.00***	-0.29 ***							
RRDA	-0.41 ***	0.18 ***	***0.04***	***-0.07***	0.83 ***	0.90 ***	-0.40 ***	0.23 ***	***0.07***	-0.11 *	0.90 ***						
10-year measurement																	
RW	0.93 ***	0.43 ***	0.55 ***	0.34 ***	-0.32 ***	-0.32 ***	0.96 ***	0.47 ***	0.60 ***	0.42 ***	-0.35 ***	-0.35 ***					
RDP	0.41 ***	0.87 ***	0.74 ***	***0.05***	0.20 ***	0.17 **	0.45 ***	0.95 ***	0.81 ***	***0.05***	0.22 *	0.21 ***	0.48 ***				
RDA	0.54 ***	0.75 ***	0.88 ***	0.31 ***	***0.01***	***0.05***	0.57 ***	0.81 ***	0.95 ***	0.34 ***	***0.00***	***0.07***	0.60 ***	0.86 ***			
RDS	0.39 ***	***0.08***	0.38 ***	0.80 ***	-0.27 ***	-0.11	0.41 ***	***0.08***	0.40 ***	0.90 ***	-0.29 ***	-0.12 *	0.43 ***	***0.09***	0.41 ***		
RDD	-0.42 ***	0.14 **	***-0.05***	-0.25 ***	0.83 ***	0.75 ***	-0.42 ***	0.17 **	***-0.04***	-0.33 ***	0.89 ***	0.82 ***	-0.41 ***	0.21 ***	***0.00***	-0.32 ***	
RRDA	-0.40 ***	***0.10***	***-0.01***	***-0.09***	0.68 ***	0.77 ***	-0.40 ***	0.13 *	***0.00***	-0.17 **	0.73 ***	0.85 ***	-0.39 ***	0.17 **	***0.06***	-0.13 *	0.89 ***

Pearson correlation coefficient was given to represent the correlation between two variables. Significant correlations indicated by *$P < 0.05$, **$P < 0.01$, ***$P < 0.001$ based on Pearson correlation test. RW: ring width (mm yr^{-1}); RDP: resin duct production (no. yr^{-1}); RDA: resin duct area (mm^2 yr^{-1}); RDS: resin duct size (mm^2 yr^{-1}); RDD: resin duct density (no. mm^{-2} yr^{-1}); RRDA: relative resin duct area (% yr^{-1}).

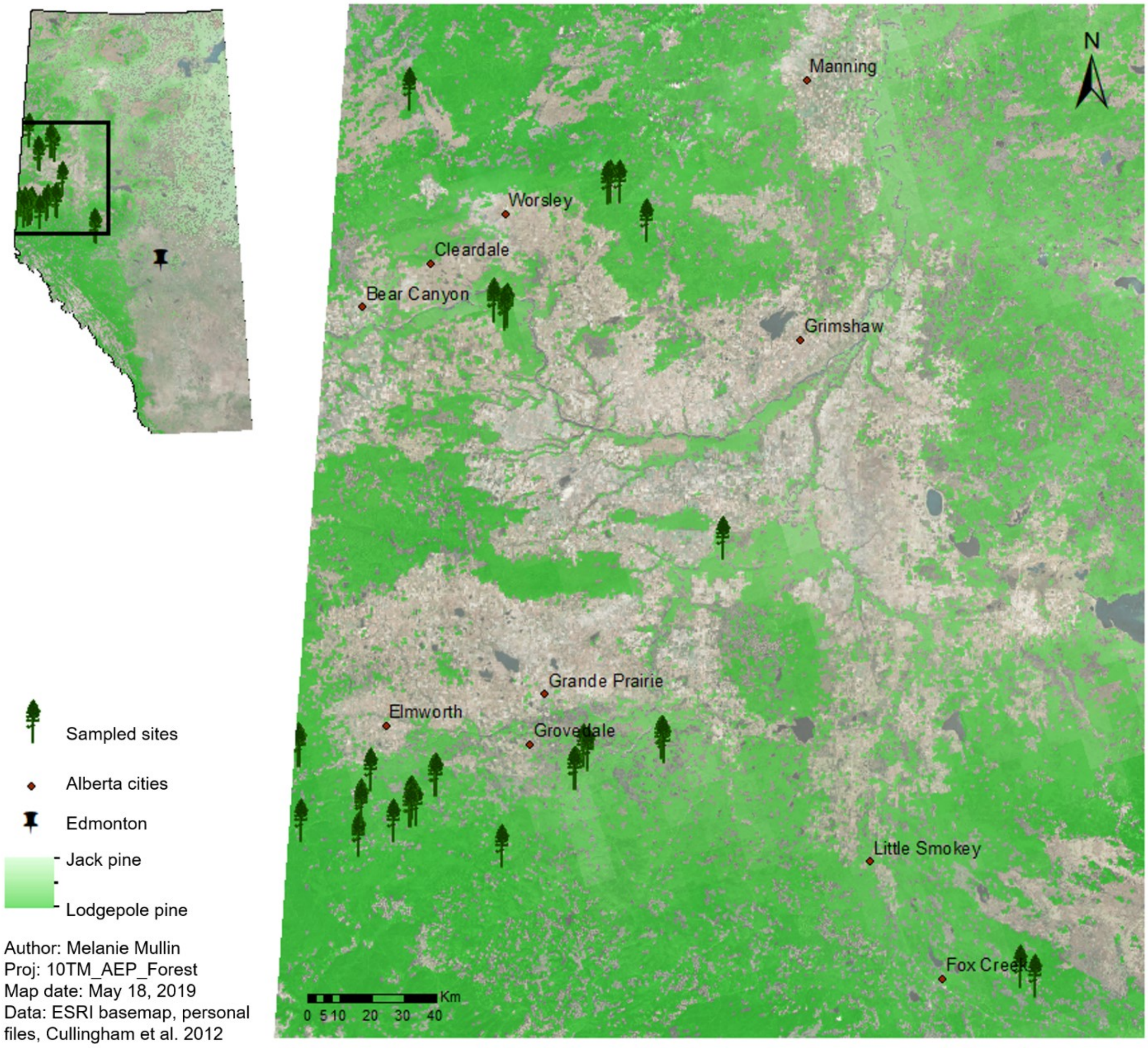

Figure S1. Geographic distribution of sites sampled for increment cores and wedges taken from *Pinus contorta* var. *latifolia* trees. Some sites were clustered together due to short distance between them; thus, the number of sites shown on the map does not reflect the actual number of sites sampled. Resource of base map was from personal files in Cullingham et al. 2012.

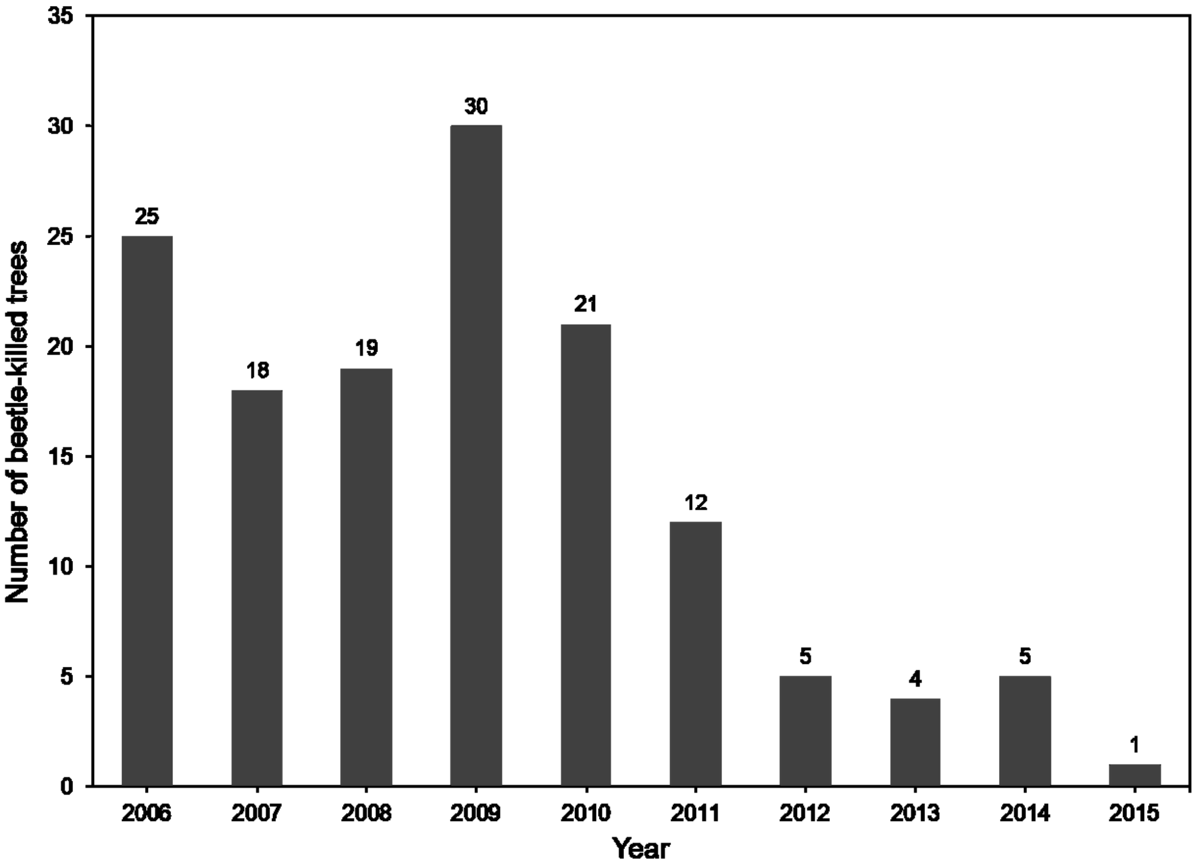

Figure S2. Annual number of sampled *Pinus contorta* var. *latifolia* trees killed by *Dendroctonus ponderosae* in selected study sites from 2006 to 2016. The total number of trees killed was 140. The death year of each sampled tree was determined by visual crossdating. The majority of trees (89%) were killed between 2006 and 2011.

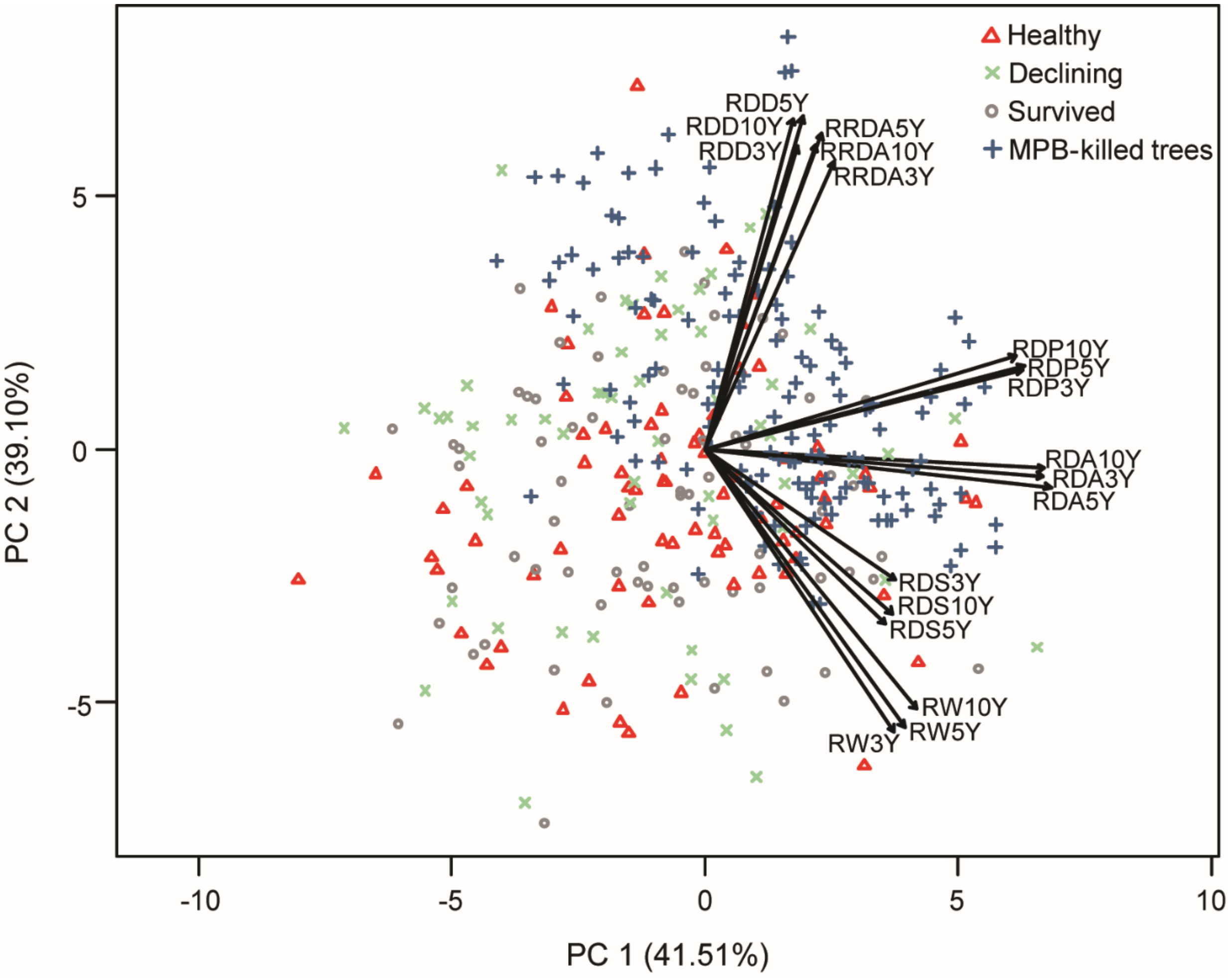

Figure S3 Results of principle component analysis (PCA) among healthy, declining, survived and mountain pine beetle (*Dendroctonus ponderosae*, MPB)-killed *Pinus contorta* var. *latifolia* trees in terms of the resin duct characteristics and ring width. Healthy trees are represented as red triangles, declining trees as green ×, survived trees as gray circles, and MPB-killed trees as blue +. Vectors indicate the strength and directional influence of resin duct characteristics and growth under deducted dimensions. RW: ring width (mm yr^{-1}); RDP: resin duct production (no. yr^{-1}); RDD: resin duct density (no. mm^{-2} yr^{-1}); RRDA: relative resin duct area (% yr^{-1}); RDA: total resin duct area (mm^2 yr^{-1}); RDS: resin duct size (mm^2 yr^{-1}). 3Y: mean of resin duct and growth characteristics in 3-year pre-outbreak growth period; 5Y: mean of resin duct and growth

characteristics in 5-year pre-outbreak growth period; 10Y: mean of resin duct and growth characteristics in 10-year pre-outbreak growth period.

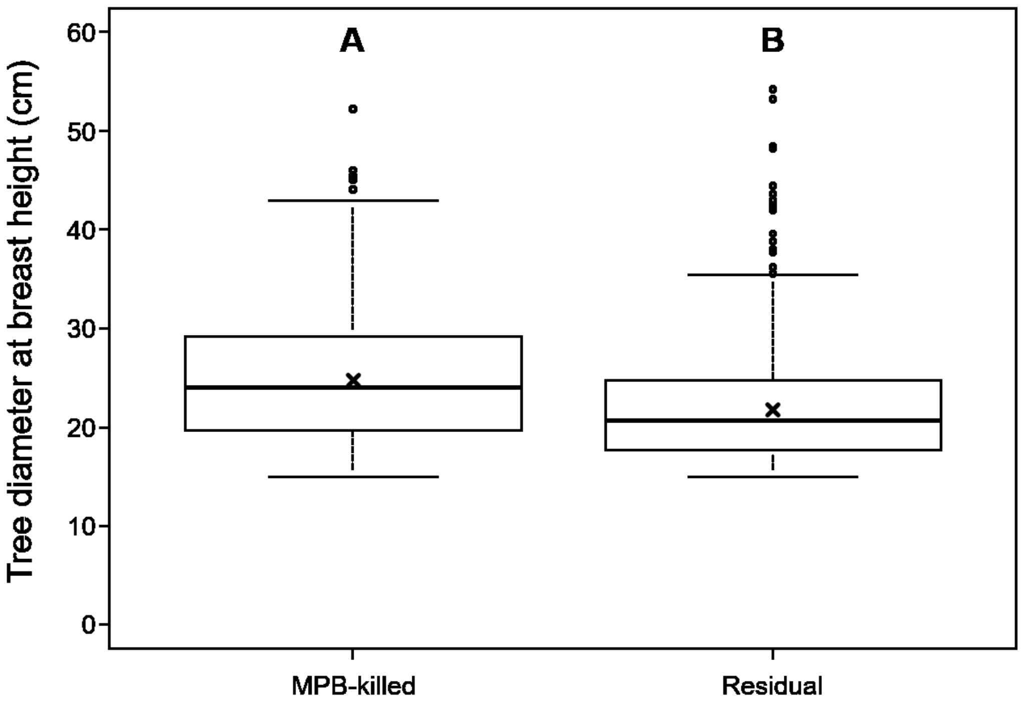

Figure S4. Tree diameter at breast height of mountain pine beetle (MPB, *Dendroctonus ponderosae*)-killed and residual *Pinus contorta* var. *latifolia* trees with bark attached in post-outbreak stands. Significant differences among the two categories of trees were indicated by different letters based on the results of linear mixed-effects models (α=0.05). In each box plot, the heavy line is the median, the × mark indicates the mean, the box represents the 1st and 3rd quartiles, whiskers are Tukey's 1.5 interquartile range, and the circles indicate outliers.
